# Acute pancreatitis associated with Cushing syndrome – A case report and literature review

**DOI:** 10.1016/j.amsu.2021.102260

**Published:** 2021-03-29

**Authors:** M. Bouali, S. Kabura, A. El Bakouri, K. El Hattabi, F.Z. Bensardi, A. Fadil

**Affiliations:** a Service of Emergency of Visceral Surgery, Ibn Rochd-Casablanca University Hospital Center, Morocco; b Department of General Surgery, Ibn Rochd-Casablanca University Hospital Center, Morocco; c Hassan 2 University of Casablanca, Morocco

**Keywords:** Acute pancreatitis, Cushing's disease, Cushing's syndrome, Corticosteroid therapy, Cushing syndrome complications

## Abstract

**Introduction:**

Cushing's syndrome (CS) is a rare and severe disease. Acute pancreatitis is the leading cause of hospitalization. The association of the two disease is rare and uncommon. We report the case of a 37-year-old woman admitted in our service for acute pancreatitis and whose Cushing syndrome was diagnosed during hospiatilisation. The aim of this work is to try to understand the influence of de Cushing in acute pancreatitis and to establish a causative relationship between the two diseases.

**Observation:**

It is a 37-year-old woman with a history of corticosteroid intake for six months, stopped three months ago who consulted for epigastralgia and vomiting. The physical exam found epigastric sensitivity with Cushing syndrome symptoms. A CT scan revealed acute edematous-interstitial pancreatitis stage E of Balthazar classification. 24 h free cortisol of 95 μg/24 h and cortisolemia of 3.4 μg/dl. The patient was treated symptomatically and referred after to endocrinology service for further treatment.

**Conclusion:**

The association with acute pancreatitis and CS is rare and uncommon. Although detailed studies and evidence are lacking, it can therefore be inferred that CS is one of the risk factors for the onset of acute pancreatitis. The medical treatment and management of acute pancreatitis in those patients do not differ from other pancreatitis of any etiologies.

## Highlights

• There is a highs suspicion of acute pancreatitis complications for patients with Cushing syndrome.
• Corticosteroids are a common cause for both Cushing syndrome and acute pancreatitis.
• There are many common etiologies between Cushing syndrome and acute pancreatitis.
• Cushing syndrome is a risk factor of acute pancreatitis, need further detailed studies.

## 1 Introduction

Cushing's syndrome (CS) is a rare and severe disease that may be secondary to prolonged treatment with glucocorticoids or to an ACTH-secreting pituitary or ectopic (lung) tumor causing excess cortisol in Cushing's disease [1]. Acute pancreatitis associated with the administration of corticosteroids has been reported as a rare but severe disease [2]. Acute pancreatitis **is not known as a complication** of Cushing syndrome. The association of the two diseases is rare and uncommon [3]. We report the case of a 37-year-old woman hospitalized in our department with **acute pancreatitis stage E no lithiasis** with Cushing syndrome discovered during hospitalization**. The aim of our study is to establish a causative relationship between CS and acute pancreatitis and to study the pathophysiology during association** of the two diseases. **This manuscript has been reported in line with SCARE's 2020 Criteria** [4].

## 2 Observation

**Fig. 1:**
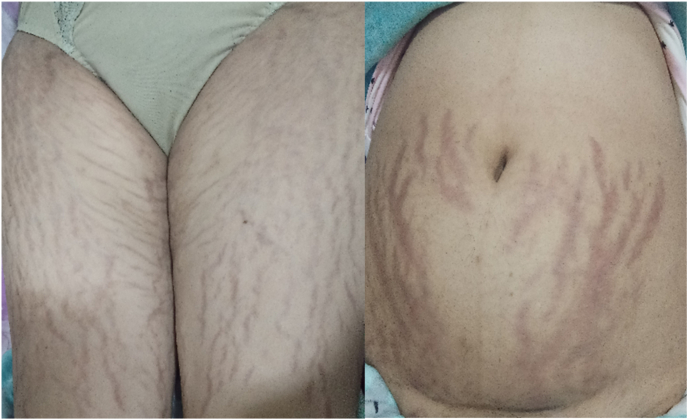
Purplish stretch marks on the lower limbs and abdomen with muscles amyotrophy. (For interpretation of the references to colour in this figure legend, the reader is referred to the Web version of this article.)

**Fig. 2:**
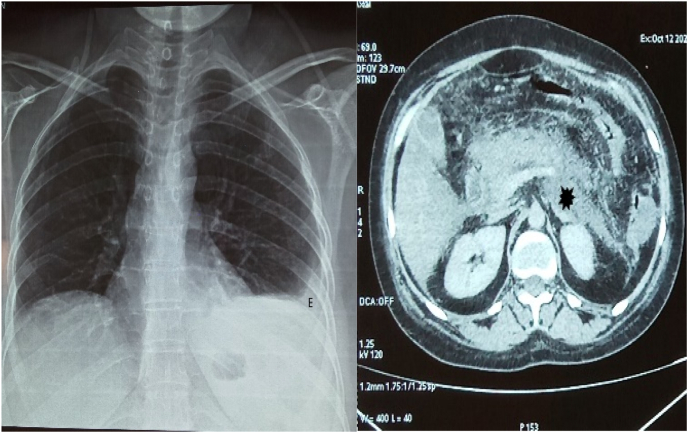
Thoracic X-ray note the pleural effusion (E) and CT scan showing a stage E acute pancreatitis with necrosis (black asterisk).

This is a 37-year-old woman **consulted our service for epigastralgia and vomiting over 48 hours. The past medical history noticed a long-term corticosteroid intake for six months for weigh**t gaining, stopped three months ago. There was no report on **change in bowel habit nor hematemesis, nor rectal bleeding**, no fever and the patient has a good overall health. The **physical examination** found a **patient conscious with GCS of 15/15**, blood pressure: 12/7 cm Hg, 75 of pulse, respiratory rate: 16 cpm, with haemodynamic and respiratory stability, **no jaundice of conjunctiva** and mucosa. **There was muscles hypotrophy of** the lower limbs, purplish striae **marks on the abdomen and lower limbs which appeared after corticosteroids intake according to medical record (**Fig. 1**) and epigastric pain.** Pelvic examination was normal. The initial biological assessment showed lipasemia: 236 IU/L; Hg: 14.8g/dl wbc: 11,460/mm; platelets: 165,000/mm; Urea: 0.12 g/l; Serum creatinine: 7.6 mg/l; CRP: 120 mg/l. 24h. Further investigations found free cortisol: 95 μg/24h (10–5095 μg/24h) cortisolemia: 3.4 μg/dl (6.4–22.8 95 μg/dl). Cushing syndrome was then diagnosed during hospitalization. An abdominal CT scan was performed showing an appearance of acute edematous-interstitial pancreatitis classified as Balthazar's E stage complicated by partial thrombosis of the splenic vein (Fig. 2). The additional ultrasound showed a semi-full gall bladder, alithiasis (Fig. 2). Chest X-ray revealed a left pleura effusion. The patient was admitted in our service **for acute pancreatitis** and received symptomatic **treatment consisting in digestive rest, rehydration** with normal saline and G5% 3l/day, parenteral nutrition with oliclinomel, analgesic, proton pump inhibitor (PPI) inexium 40mg. The recovery was obtained on 21st day with normal feed and the patient was discharged from hospital on D23 and was referred to endocrinologists for further treatment and surveillance. **She was seen in consultation after 4 months and was in good health without any treatment except the surveillance by endocrinologists.**

## 3 Discussion

Cushing's syndrome (CS) is a rare and severe endocrine disease characterized by a variety of typical signs and symptoms resulting of excess cortisol. Its prevalence is 2–3 cases per million people per year. Patients with CS may have history weight increase, weakness, delayed wound healing, bruising, bone pain, depression [5]. The physical examination found an increase in fatty deposits in the upper half of the body giving an appearance of “bison hump”, lunar facies (cushingoid facies), amyotrophy of the limbs, hirsutism, shoulder muscles weakness and hip belt, thin skin, abdominal pain, purplish stretch marks. The 24-hour urinary cortisol assay detect endogenous overproduction of cortisol. Cushing's syndrome is confirmed when result is more than four times the normal [6]. Measurement of plasma ACTH levels helps to differentiate ACTH-dependent CS from ACTH-independent CS. If the ACTH levels are consistently> 15 pg/ml, the CS is ACTH dependent. When ACTH levels are <5 pg/ml on several occasions, the CS is ACTH independent. The high dose dexamethasone suppression test (HDDST) is also used; 8 mg of dexamethasone is administered overnight. The proposed threshold for a positive response is a decrease in serum cortisol to 50% of basal level. The CRH test is used for the direct assessment of pituitary ACTH reserve. Most patients with CD respond to this test, whereas patients with ectopic ACTH syndrome are generally insensitive. The sensitivity and specificity of the test are 80% and 95%, respectively, using the ACTH response, and 91% and 95%, respectively, using the cortisol response. Pituitary MRI, CT scan of the adrenals, chest X-ray, and thoraco-abdominal CT scan are also helpful in locating pathology [7]. CS is divided into ACTH dependent CS in 10–80% of cases and ACTH independent CS. Pituitary CS or Cushing's disease (CD) is the most common etiology of endogenous CS, accounting for approximately 70–80% of cases of endogenous hypercortisolism. In CD, excess cortisol results from hypersecretion of ACTH from a pituitary tumor, adenoma in about 90% of cases or exceptionally carcinoma [1]. ACTH independent CS is caused by benign or malignant adrenal tumors, bilateral adrenal hyperplasia. Ectopic ACTH syndrome (EAS) accounts for 20% of ACTH-dependent SCs and about 10% of all CS types. It is associated with a variety of malignant tumors, mainly of neuroendocrine origin. Other tumors associated with EAS are small cell lung carcinoma, pheochromocytoma, medullary thyroid carcinoma, and carcinoma of the prostate [8]. The body alteration due to hypercorticism (obesity, tissue fragility, sensitivity to infections, muscle wasting, diabetes, atheroma, hypertension, etc.) therefore appears to be an essential factor in the onset of acute pancreatitis. Clague et al. [9] have reported a case of acute pancreatitis complicating ectopic ACTH secretion syndrome in the absence of adrenal surgery; two experimental studies in rats have shown the direct role of cortisolic hypersecretion during acute pancreatitis induced in adrenalectomized rats [10]. It has recently been reported that the plasma levels of certain cytokines are elevated in patients with pancreatitis, and that these can predict its severity and the onset of complications. A recent experimental study suggested that increased tumor necrosis factor (TNF) production may play a role in the occurrence of systemic complications in acute pancreatitis. Therefore, it is interesting to study the role of the hypothalamic-pituitary-adrenal axis in the occurrence of acute pancreatitis [11]. Acute pancreatitis is the leading cause of hospitalization for gastrointestinal disorders in many countries with an annual incidence ranges from 13 to 45 cases per 100,000 people and a global estimate of 33.74 cases per 100,000 people, causing a health problematic worldwide [12]. The mortality is <1% for the mild form, but which can reach 30% for the severe forms, in particular in cases of infection of necrosis collections and multi-visceral failure. The main etiologic factors are gallstones and alcohol [13]. Other risk factors are genetic predisposition, drugs, smoking, hypertriglyceridemia, type 2 diabetes and endoscopic retrograde cholangiopancreatography [14]. Drugs are responsible for 0.1%–2% of incidents of acute pancreatitis [15]. Some studies have suggested a dose-dependent risk of developing acute pancreatitis during treatment with corticosteroids, with thresholds of 25 mg/day for prednisolone [2,16]. Rare cases of acute pancreatitis have been reported after left adrenalectomy for Cushing's syndrome, Cushing's disease or ectopic ACTH secretion syndrome, although the occurrence of this complication is exceptional [10]. Due to the rarity of cases, it is difficult to establish from a series of studies a relationship between pancreatitis and corticosteroids or CS, especially while in the literature, cases of acute pancreatitis induced by corticosteroid without CS are reported and CS corticosteroid-induced without pancreatitis are observed. By the way, no study has been able to demonstrate the relationship between pancreatitis and Cushing's disease. **This case illustrates the possibility of acute pancreatitis complicating Cushing syndrome. It has the limits that it is only for one case report. Further studies are necessary as series to confirm this.** The severity and outcome of AP are influenced by the patient's metabolic comorbidities. Cushing syndrome treatment is essential to reduce mortality, improve comorbidities and long-term quality of life. Surgical resection of the causative lesions is the ideal and most effective treatment for normalizing cortisol secretion. Radiotherapy for corticotropic tumors is the second-line treatment. The choice of these treatments is complex, must be carried out by a team of multidisciplinary experts to be individualized for each patient, and use a shared decision-making approach [12,17]. It is multidisciplinary, bringing together emergency doctors, gastroenterologists or visceralists [18]. Hydroelectric rehydration to prevent hypovolemia and organ hypoperfusion is a cornerstone in the treatment of acute pancreatitis. Prophylactic antibiotic therapy is discussed due to the frequency of infection of necrosis collection [19]. The medical treatment and management of acute pancreatitis in a patient with Cushing's syndrome is similar to that of pancreatitis of other etiologies.

## 4 Conclusion

Cushing's syndrome (CS) is a rare and severe disease caused by prolonged treatment with glucocorticoids (GC) or by an ACTH-secreting tumor responsible for excess cortisol. Glucocorticoids are responsible for both CS and acute pancreatitis according to data in the literature. In addition, among the complications of CS is the metabolic syndrome which is also implicated in the occurrence of acute pancreatitis. Thus, although detailed studies and evidence are lacking, it can therefore be inferred that CS is one of the risk factors for the onset of acute pancreatitis. However, further studies are needed to establish the pathophysiology because one case study cannot confirm the causative relationship between the two diseases.

## Authors' contributions

This work was carried out in collaboration among all authors. Authors KS and MB designed the study, managed the literature researches, wrote the protocol and the first draft of the manuscript. Authors KS, MB, AEB performed the analyses of the study and. All authors read and approved the final manuscript.

## Consent

Written informed consent was obtained from the patient for publication of this case report and accompanying images. A copy of the written consent is available for review by the Editor-in-Chief of this journal on request.

## Ethical approval

As per international standard, written ethical approval has been collected and preserved by the author(s).

## Provenance and peer review

Not commissioned, externally peer-reviewed.

## Guarantor

Sylvestre KABURA.

## Declaration of competing interest

The authors declare that they have no conflicts of interest in connection with this article.
